# Prenatal Ethanol Exposure Alters Synaptic Plasticity in the Dorsolateral Striatum of Rat Offspring via Changing the Reactivity of Dopamine Receptor

**DOI:** 10.1371/journal.pone.0042443

**Published:** 2012-08-16

**Authors:** Rong Zhou, Shenjun Wang, Xuejiang Zhu

**Affiliations:** Department of Physiology, Nanjing Medical University, Nanjing, Jiangsu, China; McLean Hospital/Harvard Medical School, United States of America

## Abstract

Prenatal exposure to high-level ethanol (EtOH) has been reported to produce hyperlocomotion in offspring. Previous studies have demonstrated synaptic plasticity in cortical afferent to the dorsolateral (DL) striatum is involved in the pathogensis of hyperlocomotion. Here, prenatal EtOH-exposed rat offspring were used to investigate whether maternal EtOH exposure affected synaptic plasticity in the DL striatum. We found high-frequency stimulation (HFS) induced a weaker long-term potentiation (LTP) in EtOH rats than that in control rats at postnatal day (PD) 15. The same protocol of HFS induced long-term depression (LTD) in control group but still LTP in EtOH group at PD 30 or PD 40. Furthermore, enhancement of basal synaptic transmission accompanied by the decrease of pair-pulse facilitation (PPF) was observed in PD 30 EtOH offspring. The perfusion with D1-type receptors (D1R) antagonist SCH23390 recovered synaptic transmission and blocked the induction of abnormal LTP in PD 30 EtOH offspring. The perfusion with D2-type receptors (D2R) agonist quinpirole reversed EtOH-induced LTP into D1R- and metabotropic glutamate receptor-dependent LTD. The data provide the functional evidence that prenatal ethanol exposure led to the persistent abnormal synaptic plasticity in the DL striatum via disturbing the balance between D1R and D2R.

## Introduction

Heavy ethanol (EtOH) consumption during pregnancy has grave and multifaceted consequences for child development [1]. The most far-reaching consequence of prenatal EtOH exposure is that its effect on the brain brings the ensuing behavioral alterations [2], [3]. Numerous clinical investigations have found that children, boys in particular, exposed to EtOH in utero display hyperlocomotion, which may not be diagnosed until their educational years, and this deficit may increase in severity during the adult stage [4]–[6]. Similarly, this behavioral abnormality observed clinically could be widely mirrored by prenatal EtOH exposed animal models [7]–[10]. Although there is a considerable amount of data regarding the morphological and behavioral effects associated with prenatal EtOH exposure [11], the mechanisms underlying developmental defects caused by maternal EtOH consumption remain unclear.

The striatum, especially the dorsolateral (DL) subregion, appears to play a critical role in assisting voluntary motor behaviors in humans, other primates and rodents [12], [13]. The neocortex provides major glutamatergic inputs to striatal medium spiny projection neurons. Plasticity at corticostriatal synapses is thought to provide a cellular basis for stiratum-dependent behaviors [14], [15]. Two forms of synaptic plasticity can be induced by high frequency stimulation (HFS) in the DL striatum: long-term depression (LTD) and long-term potentiation (LTP) [16]–[20]. Partridge et al. (2000) have reported that in the developing rat DL striatum, the conversion from LTP to LTD occurs during the period of the postnatal 3rd week [21]. Morphological and electrophysiological properties of striatal neurons have been reported to become mature after the postnatal 3rd weeks [22]. Until recently, few experiments have been conducted to study whether prenatal EtOH exposure affects synaptic plasticity of the postnatal developing DL striatum.

Dopaminergic projection from midbrain nuclei is another important input in striatum. Earlier studies on the striatum have shown that dopamine system participates in the change of striatal synaptic plasticity during the early postnatal development via both pre- and post-synaptic mechanisms [23]. Dopamine-mediated action is achieved by its interaction with two types of G protein-coupled receptor, D1-type receptors (D1 or D5, termed here D1R) and D2- type receptors (D2, D3 or D4, termed here D2R) [24], [25]. The down-regulation or knockout of D1R prevents either the induction of LTD or LTP [20], [26], [27] and reduces spontaneous motor activity [28], [29]. Interestingly, in D2R- knockout mice, HFS of corticostriatal fibers induces LTP instead of LTD [30]. Previous studies have indicated that prenatal EtOH exposure results in a long-lasting perturbation of central dopamine receptor sensitivity [5], [31]. It has been reported that prenantal EtOH exposure enhances the reactivity of D1R in the rat brain [5], [32]. Hence, we pose a hypothesis that prenatal EtOH exposure affects dopaminergic systems leading to the changes of synaptic plasticity in the DL striatum.

This study investigated whether prenatal exposure to a relatively high-dose EtOH affected synaptic plasticity in the DL striatum of rat offspring, and if so, whether the functions of dopamine receptors were involved in the alteration of synaptic plasticity and basal synaptic properties.

## Materials and Methods

### Ethics Statement

The present studies were approved by Animal Care and Use Committee of Nanjing Medical University (ID: 2008031911). The protocols used here were in accordance with the guidelines published in the NIH Guide for the Care and Use of Laboratory Animals. All efforts were made to minimize the number of animals and their suffering.

### Preparation of prenatal EtOH exposed animal model

#### Subjects

Pregnant Sprague-Dawley rats (Oriental Bio Service Inc., Nanjing) were delivered to our laboratory on gestation day (GD) 2. The morning that vaginal plugs were found was designated as GD 0, and births were expected on GD 21–22. Females were housed individually in polyethylene maternity cages (44×25×20 cm) under environmentally controlled conditions (7:00 am lights on, 7:00 pm lights off, ambient temperature at 20–23°C). The pregnant dams and their offspring were monitored with regard to body weight gain. The day of birth was referred to as postnatal day 0 (PD 0). The dams were allowed to nurse the own young before weaning occurred at PD 21. Litters were then culled to a maximum of 10 pups by sex. One male offspring was randomly selected from each litter as the object of study.

#### Treatment

On gestational day (GD) 7, pregnant dams were divided into control and EtOH group. Starting from GD 7 throughout GD 20, dams from EtOH group were daily administrated with 6 g EtOH/kg body weight, with ad libitum access to laboratory chow and water. Animals in control group received the same volume of isocaloric sucrose solution as EtOH. The EtOH/sucrose solution was delivered by intragastric intubations. Binge-like regime of EtOH administration was chosen as producing higher blood EtOH concentration (BEC) [33], [34] and thus being more damaging as compared to the liquid diet. To determine BECs for EtOH-treated dams on GD 20, 20 µl of blood was taken from each subject and rapidly analyzed by gas–liquid chromatography [35].

### Electrophysiological analysis

#### Slice preparation

The procedure used was similar to that described previously [21]. Animals were killed by decapitation, their brains were immediately removed and placed in ice-cold (−3°C) modified artificial cerebrospinal fluid (ACSF) containing the following substances (in mM): 124 NaCl, 2 CaCl_2_, 4.5 KCl, 1.0 MgCl_2_, 26 NaHCO_3_, 1.2 NaH_2_PO_4_, and 10 D-glucose and adjusted to pH 7.4 by bubbling with 95% O_2_/5% CO_2_. Coronal brain slices (400 µm) were cut using a vibrating microtome (Microslicer DTK 1500, Dousaka EM Co, Kyoto, Japan) in ice-cold oxygenated ACSF. The slices containing the DL striatum were stored for a minimum of 1 h prior to recording in oxygenated ACSF maintained at room temperature.

#### Field potential recording

For recording, slices were transferred to a chamber continuously perfused with oxygenated ACSF (2 ml/min) maintained at 30±1°C. Stimulation consisted of monophasic wave pulses delivered through a stainless steel electrode placed in the white matter overlying the DL striatum. The stimulation-evoked population spike (PS) was recorded from the DL striatum through glass micropipettes filled with 2 M NaCl (4–5 MΩ) connected with a differential AC amplifier (A-M Systems, model 1700, Seattle, WA). The experimental control, data acquisition, and analysis were performed using pCLAMP software (Molecular Devices, Union City, CA). The PS amplitude was defined as the average of the amplitude from the beginning to the peak negativity, and the amplitude from peak negativity to the end. Input-output (I/O) curves were constructed to examine basal synaptic transmission by delivering an ascending series of 11 stimulus intensities (0.03–0.68 mA) that ranged from sub-threshold intensity for elicitation of a PS to that eliciting maximal response. Test stimulus which induced 50% of a maximum of PS amplitude was required for the followed procedures. Paired-pulse response of PS was evoked by pair test stimulus at corticostriatal afferent fibers. The intervals between double pulses were 25 ms, 50 ms, 75 ms, 100 ms, 150 ms, 300 ms and 500 ms. To induce long-term depression (LTD) or long-term potentiation (LTP) occurring at the site of corticostriatal synapse, the test stimulus induced PS was recorded for no less than 20 min as the base level. High frequency stimulation (HFS, 4 trains, 1 s duration, 100 Hz frequency, at 10 s intervals) with 50% of maximal stimulus strength was then chosen as conditioning stimulus to be given from electrodes. The same recordings as those before HFS continued for 60 min after HFS. Each PS amplitude pre- or post-HFS was normalized with the percentage of the mean pre-HFS value. Because HFS induced small change of PS response (<20%) is not stable and persistent, the successful induction of LTD or LTP requires the decrease or increase of PS amplitude post-HFS during the stable phase (>30 min post-HFS) exceeds a minimum of 20% [36]–[38].

#### Drug administration

SCH23390, L-sulpiride, quinpirole, 2-methyl-6-(phenylethynyl)-pyridine (MPEP), d-2-amino-5-phosphonopentanoic acid (D-AP5) used in the present study were all obtained from Sigma-Aldrich (St. Louis, MO). L-sulpiride, quinpirole, MPEP, D-AP5 were dissolved to their final concentration in ACSF. SCH23390 was dissolved in ACSF containing 0.1% DMSO. Drug solutions entered the recording chamber within 40 s after a three-way tap had been turned on. They were applied in the bath for at least 30 min starting from 15 min pre-HFS.

#### Data analysis/statistics

Data were retrieved and processed with the software Micro cal Origin 6.1. The group data were expressed as the means ± standard error (SE). Experimental results were compared among treatment groups by ANOVAs followed by Bonferroni's post hoc test or t test. Statistical analysis was performed using State7 software (STATA Corporation, USA). P<0.05 was considered statistically significant. For statistical purposes, only one slice was studied per rat in electrophysiological analysis.

## Results

### Prenatal EtOH exposure analysis

The mean BECs for EtOH-treated dams were 302.89±19.5 and 331.21±28.9 mg/dl, 2 and 3 h after the last intubation on GD 20 (n = 20), respectively, suggesting binge-like EtOH administration during gestation results in maternal high-level BEC which corresponded with the previous reports [37]. Prenatal EtOH exposure had no effect on the length of gestation, average litter size, or the distribution of male and female offspring (data not shown).

### The disturbed conversion of striatal synaptic plasticity in EtOH offspring

**Figure 1:**
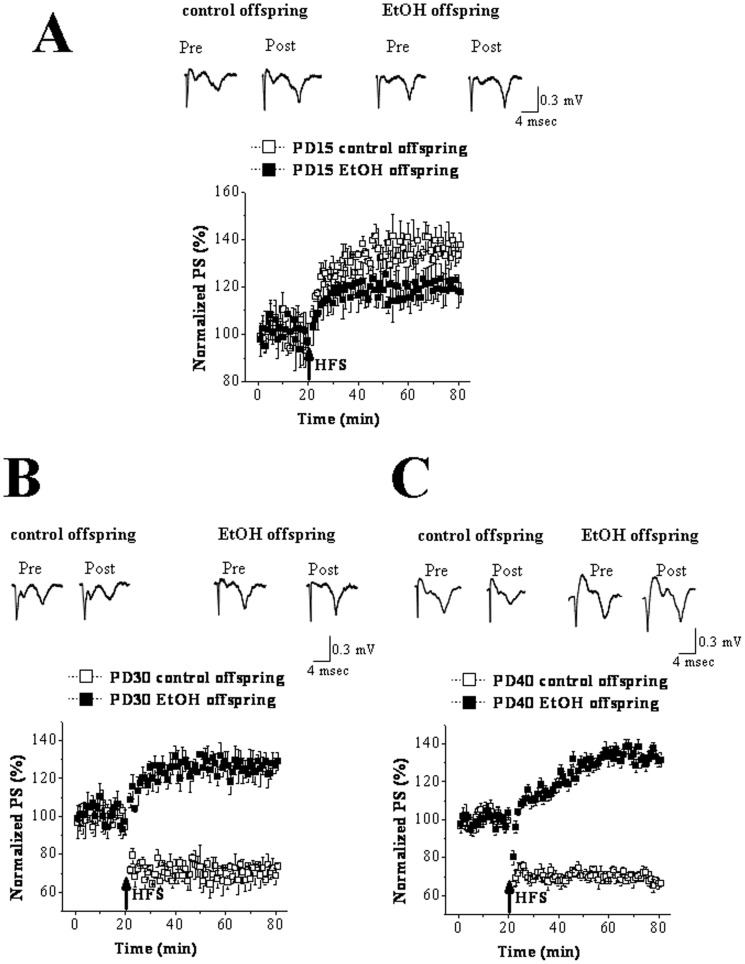
Prenatal EtOH exposure changed the postnatal synaptic plasticity at corticalstratial pathway in the DL striatum. **A:** Synaptic plasticity in PD 15 control and EtOH offspring. The upper traces are the original PS waves at 15 min pre-HFS and 60 min post-HFS. Calibration bars: 0.3 mV and 4 msec. ‘↑’ indicates the time of HFS delivery. Note that HFS induced the significant potentiaton of the PS 60 min post-HFS indicative of LTP in control offspring, while did not change the PS amplitude 60 min post-HFS in EtOH offspring. **B&C:** Synaptic plasticity in PD 30–40 control and EtOH offspring. Note that HFS induced LTD in control offspring, but LTP in EtOH offspring.

To determine whether prenatal EtOH exposure affected synaptic plasticity in the DL striatum of rat offspring, synaptic plasticity at PD 15, PD 30 and PD 40 was recorded and compared between control and EtOH offspring. As shown in Figure 1A, HFS caused a significantly persistent potentiation of PS (133.49±4.83% at 60 min post-HFS, n = 14) in PD 15 control offspring, showing a representative sample of LTP. By contrast, the same HFS protocols induced a mild increase of PS amplitude in the same-aged EtOH offspring (115.59±5.70%, n = 16). In PD 30 control offspring, HFS induced the depression of PS amplitude for over 60 min (70.67±3.89%, n = 10), indicating the successful induction of LTD (Fig. 1B). Interestingly, the same mode of HFS induced LTP but not LTD in the slices from PD 30 EtOH offspring (127.71±3.31%, n = 10; Fig. 1B). The similar results to that in Figure 1B were obtained in PD40 control and EtOH offspring (control group: 67.63±2.92%, n = 10; EtOH group: 130.58±2.80%, n = 10; Fig. 1C). In addition, there was no significant difference in the size of striatum between control and EtOH offspring at any of three postnatal ages (data not shown). Consistent with the present findings about control rats, Partridge et al. (2000) have reported that LTP is the dominating form during PD12–14, while LTD replaces LTP during PD24–32 in the developing rat DL striatum [21]. The results obtained from EtOH offspring suggested the possibility that prenatal EtOH exposure led to the impairment of reversal development of synaptic plasticity in the DL striatum. It has been reported that adult-typical behaviors of rats are completely established during PD21–23 [32]. Hereafter, we paid great attention on the mechanisms underlying HFS-induced LTP instead of LTD in the DL striatum of PD 30 EtOH offspring.

### Enhancement of basal synaptic transmission with the increase in presynaptic glutamate release at corticostriatal pathway of EtOH offspring

**Figure 2:**
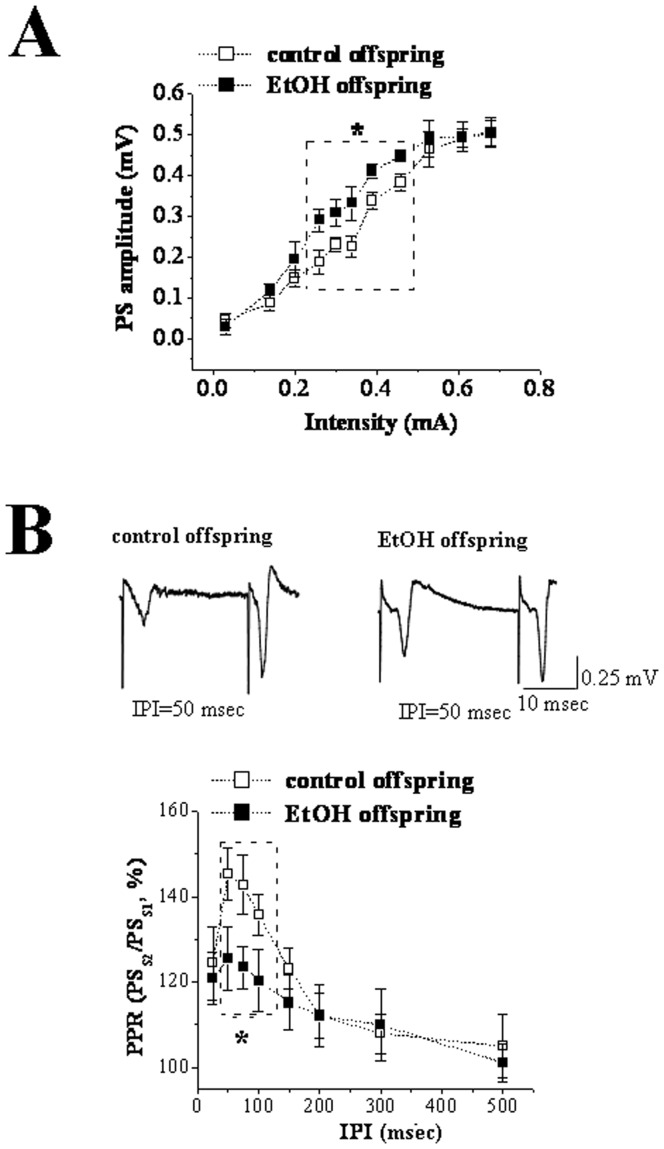
Prenatal exposure EtOH potentiates basal synaptic transmission of PD 30 offspring via increasing presynaptic glutamate release of corticostriatal pathway. A: I/O curve in control and EtOH offspring. Note that the amplitude of PS elicited by 0.26–0.46 mA test stimulus in EtOH offspring is higher than that in control rats. *, P<0.05. **B:** PPR plotted against IPIs from25 to 500 msec in PD 30 control and EtOH rats. The upper figure is the original data of PPR with IPI at 50 msec. Calibration bars: 0.25 mV and 10 msec. Note that PPR corresponding to 50–150 IPIs in EtOH offspring is significantly lower than that in control rats. *, P<0.05.

I/O curves were constructed to identify whether prenatal EtOH exposure influenced basal synaptic transmission in striatum. Two-way ANOVA analysis indicated statistically significant effects with EtOH exposure (F(1,22) = 10.481; P = 0.001), stimulation intensity (F(1,22) = 65.812; P<0.00001; Fig. 2A). Post-hoc analysis using Bonferroni's test further revealed PS amplitudes in EtOH offspring were significantly higher than those in control offspring at the same stimulation intensity when stimulation intensity ranged from 0.26 mA to 0.46 mA (control group: n = 10, EtOH group: n = 12; P<0.05). At higher or lower stimulation intensity, there was no significant difference in PS amplitude between two groups (P>0.05). Paired-pulse facilitation (PPF) was introduced to detect whether presynaptic glutamate release participates in the potentiation of basal synaptic transmission in EtOH rats. PPF is well known as a special phenomenon that the second stimulus evoked PS with enlarged amplitude with paired-pulse stimulation of striatum. PPF is always expressed as the ratio of the second PS amplitude to the first one and regarded to have negative correlation with presynaptic glutamate release. The data of PPF were collected corresponding to paired-pulse stimulation with different inter-pulse intervals (IPIs) from 25 to 500 msec. As shown in Figure 2B, two-way ANOVA analysis on PPF revealed significant effects with EtOH exposure (F(1,22) = 10.744; P = 0.001) and IPIs (F(1,22) = 10.188; P<0.00001). Bonferroni's post-hoc test further indicated PPF in EtOH rats was significantly less than that in control rats at the same IPIs when IPIs ranged from 50 msec to 100 msec (control group: n = 10, EtOH group: n = 12; P<0.05). Taken together, the findings indicate that the potentiation of basal synaptic transmission in PD 30 EtOH offspring is, at least part, due to the increase in presynaptic glutamate release.

### Up-regulation of D1R function is involved in EtOH-induced potentiation of basal synaptic transmission

**Figure 3:**
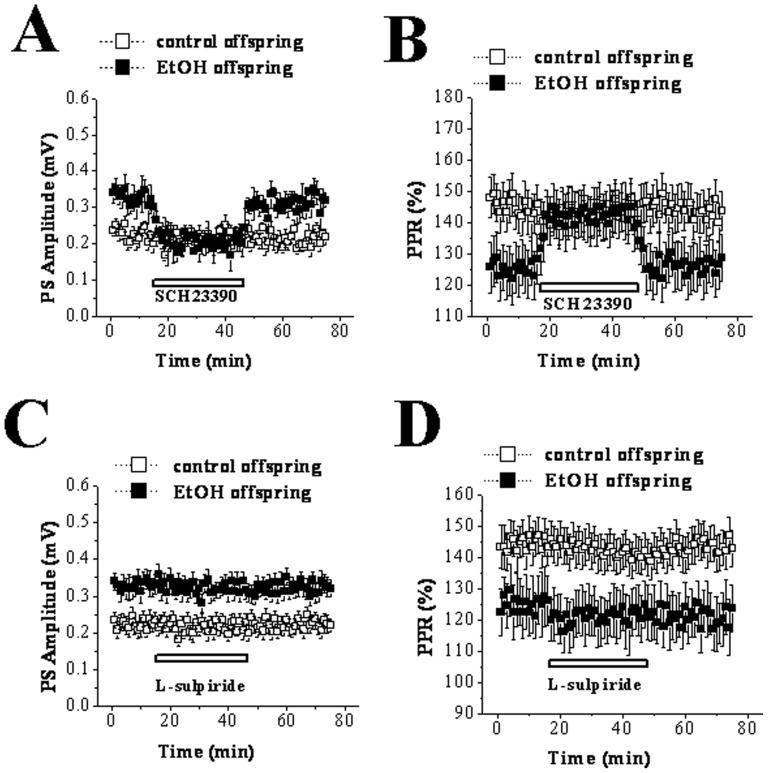
Dopamine receptors are involved in EtOH induced potentiation of basal synaptic transmission at corticostratial pathway in PD 30 offspring. The hollow line represents the duration of drug perfusion. **A & B:** Effect of the D1R antagonist SCH23390 on test stimulus evoked PS and PPF in control and EtOH rats. Note that SCH23390 inhibits the potentiation of PS and the reduction of PPF in EtOH rats, but did not affect those in control rats. **C & D:** Effect of the D2R antagonist L-sulpiride on test stimulus evoked PS and PPF in control and EtOH rats. Note that the treatment with L-sulpiride had no influence on PS amplitude or PPF at 50 msec IPI in both groups.

Earlier studies have reported that D1R activation improves the presynapse release of glutamate, while D2R activation plays an opposite effect [39], [40]. The D1R antagonist SCH23390 and the D2R antagonist L-sulpiride were applied to examine whether dopamine receptors were involved in the potentiation of basal synaptic transmission induced by prenatal EtOH exposure. The findings in Figure 3A showed that the perfusion with SCH23390 (10 µM) for 30 min did not affect the amplitude of test stimulus-evoked PS in the slices obtained from PD 30 control rats (basal, 0.22±0.05 mV; SCH23390, 0.21±0.07 mV; n = 11; t = 0.093; P = 0.928; paired t test). However, SCH23390 completely abolished the increase of test stimulus-evoked PS amplitude in PD 30 EtOH rats (basal, 0.31±0.06 mV; SCH23390, 0.20±0.04 mV; n = 12; t = 4.383; P = 0.001; paired t test). Similarly, the treatment with SCH23390 recovered the reduction of PPR evoked by pair-pulse stimulation with 50 msec IPI in EtOH offspring (n = 12; t = 4.986; P = 0.000001; paired t test) without changing that in control rats (n = 11; t = 0.318; P = 0.757; paired t test; Fig. 3B). However, the perfusion with 10 µM L-sulpiride for 30 min had no significant effect on either PS amplitude or PPR in control and EtOH group (Fig. 3C, 3D). In addition, application of 0.1% DMSO (the vehicle of SCH23390) alone did not affect PS amplitude in either control or EtOH offspring (data not shown). These data suggest that prenatal EtOH exposure results in the increase of glutamate release probably *via* up-regulation of D1R function, which, in turn, brings the potentiation of basal synaptic transmission.

### Up-regulation of D1R function LTP participates in LTP instead of LTD in PD 30 EtOH offspring

**Figure 4:**
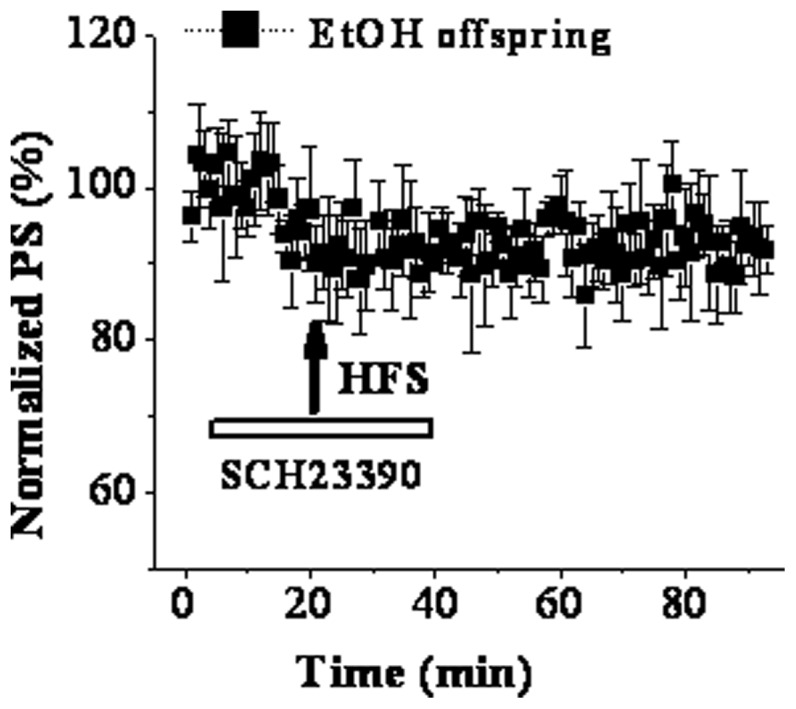
Up-regulation of D1R participates in the facilitation of LTP in PD 30 EtOH rats. ‘↑’ indicates the time point of HFS application. The hollow line represents the period of drug occurrence in ASCF. Note that SCH23390 completely blocked the potentiation of PS amplitude at 60 min post-HFS.

In order to further investigate whether up-regulation of D1R function participated in the conversion from LTD to LTP, corticostriatal synaptic plasticity was then induced in presence of SCH23390. The findings showed that SCH23390 completely blocked the LTP induction, but failed to make it return to LTD (92.15±6.43%, n = 8; Fig. 4). This result suggests D1R up-regulation is one of the possible mechanisms underlying the appearance of LTP instead of LTD in PD 30 EtOH offspring.

### Down-regulation of D2R function blocks LTD induction in PD 30 EtOH rats

**Figure 5:**
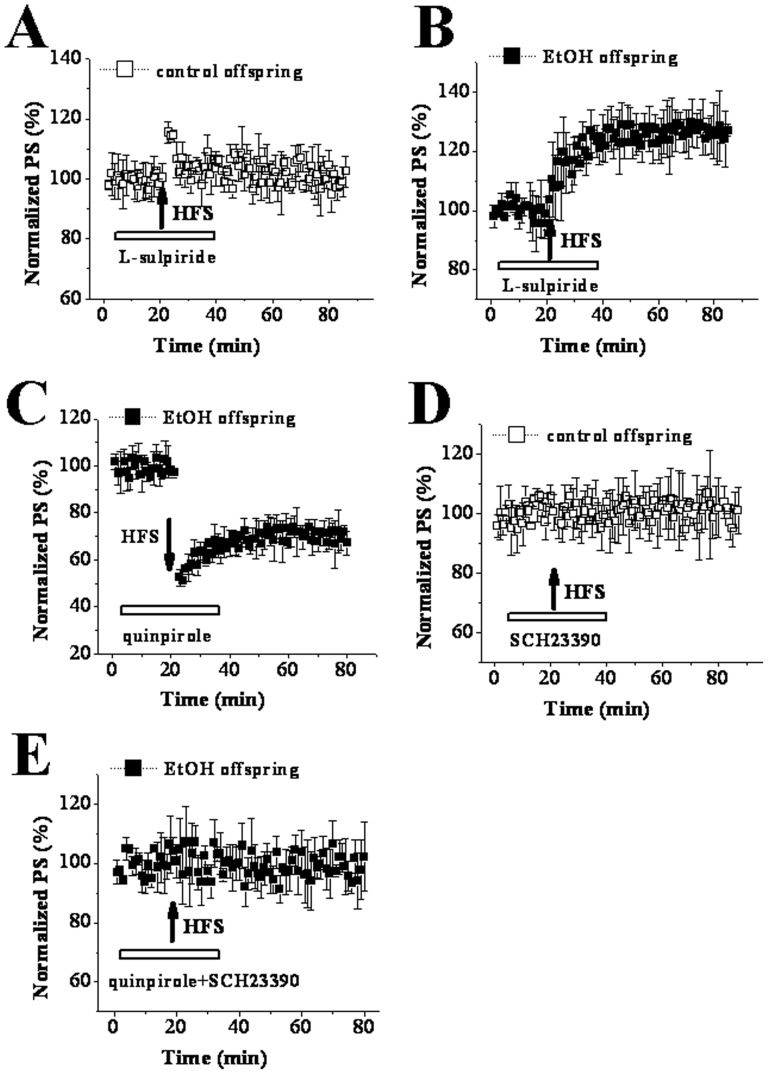
Down-regulation of D2R leads to the shift of synaptic plasticity from LTD to LTP in PD 30 EtOH rats. ‘↑’ indicates the time point of HFS application. The hollow line represents the period of drug occurrence in ASCF. **A & B:** L-sulpiride completely blocked HFS induced LTD in control rats, but had no effect on HFS induced LTP in EtOH rats. **C:** the D2R agonist quinpirole recovered the induction of LTD in EtOH rats. **D & E:** SCH23390 completely blocked either the induction of LTD in control rats or quinpirole-recovered LTD in EtOH rats.

A large number of documents indicate that D2R, as well as D1R, plays an important role in striatal synaptic plasticity. Calabresi et al. (1997) have found that HFS at corticostriatal fibers induces LTP instead of LTD in D2R knockout mice [30]. This part of our study was performed to make clear whether D2R activation mediated the conversion from LTP to LTD in EtOH offspring. The perfusion with 10 µM L-sulpiride for 30 min blocked HFS induced LTD, but did not reverse it to LTP in control slices (100.06±5.18%, n = 8; Fig. 5A). The same treatment with L-sulpiride did not alter LTP measured in EtOH offspring (125.45±6.45%, n = 8; Fig. 5B). However, the application of quinpirole (10 µM), a D2R agonist, revealed the similar LTD in EtOH offspring with that in control offspring (71.21±4.67%, n = 8; Fig. 5C). In control offspring the induction of LTD was sensitive to SCH23390 (99.46±7.50%, n = 12). Similarly, the quinpirole-recovered LTD in EtOH offspring was also blocked by SCH23390 (98.07±6.82%, n = 10; Fig. 5D).

**Figure 6:**
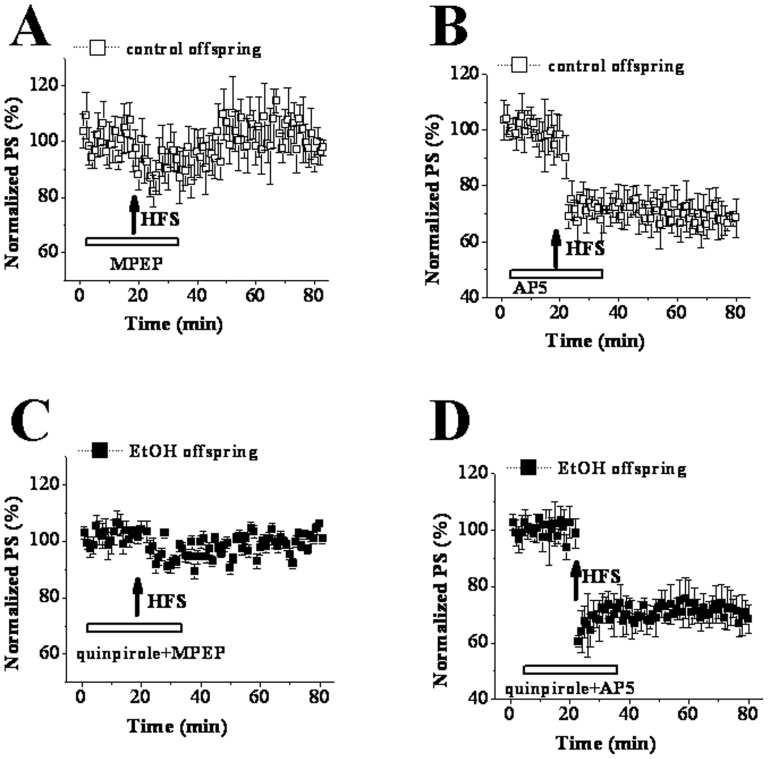
mGluR activation mediates the quinpirole-recovered LTD in EtOH rats. ‘↑’ indicates the time point of HFS application. The hollow line represents the period of drug occurrence in ASCF. **A & B:** the LTD in control rats was sensitive to MPEP, but not AP5. **C & D:** The quinpirole-recovered LTD in EtOH rats was inhibited by MPEP, but not AP5.

Previous studies have reported that metabotropic glutamate receptor (mGluR) rather than N-methyl-D-aspartate receptor (NMDAr) is involved in HFS-LTD induction in striatum, [17], [18], [41]. Our results revealed that in control offspring this LTD was blocked by the mGluR antagonist MPEP (10 µM) (97.83±2.82%, n = 10; Fig. 6A), but not the NMDAr antagonist AP5 at 50 µM (68.43±6.87%, n = 8; Fig. 6B). In EtOH offspring the quinpirole-rescued LTD was also sensitive to MPEP (103.23±2.03%, n = 10; Fig. 6C) but not AP5 (70.74±4.25%; Fig. 6D). The findings indicate that D2R down-regulation together with D1R up-regulation participates in a profound shift in the direction of long-term change at corticostriatal synapses (NMDAr-dependent LTP instead of mGluR-dependent LTD) in PD 30 EtOH offspring.

## Discussion

This study represents the first functional demonstration that prenatal exposure to a relatively high-dose EtOH leads to the persistent emergence of abnormal LTP instead of LTD *via* altering D1R and D2R functions in the DL striatum of young male rat offspring. To support this conclusion we provided the major observations as follows. First, PD 30 or PD 40 control offspring expressed LTD, while the same-aged EtOH offspring showed LTP instead of LTD. Second, in comparison with control offspring, EtOH offspring showed a D1R-mediated potentiation of basal synaptic transmission through increasing presynaptic glutamate release. Third, D1R antagonist SCH23390 blocked the induction of LTP in EtOH-exposed offspring. Fourth, D2R agonist quinpirole could rescue the D1R- and mGluR-dependent LTD induction in EtOH-exposed offspring.

The present study found that LTP was induced in the DL striatum of PD 15 control rats, while LTD was elicited in PD 30 or PD 40 control rats by the same HFS protocol. Consistent with our results, Partridge et al. (2000) have reported that in the developing rat striatum the conversion from LTP to LTD occurs during the postnatal third week [21]. Tepper and associates (1998) have shown that the third week of postnatal maturation in rat striatum is an intense period of electrophysiological and morphological change [22]. This discriminating pattern of synaptic development may give rise to functional differences in integrating afferent input during this stage of striatal development. LTP is a predominant form of plasticity when synapses are beginning to come on-line in striatum [21]. The emergence of LTD later in development is thought to help to fine-tune synaptic efficacy to refine movement and behavioral sequencing [16], [42]. This is in line with the fact that LTD predominates during a time period when movement patterns are changing from more neonatal to more adult-like [21]. LTP appearing in the early development has been considered to depend on NMDAr activation [21]. Studies in the review by Costa et al. (2000) have shown prenatal EtOH exposure led to a decrease in either NMDAR expression or function in various regions of the brain [43]. Therefore, we speculated the impairment of LTP in PD15 EtOH-exposed offspring might be the consequence of NMDAr down-regulation. Furthermore, in the present study, prenatal EtOH exposure resulted in LTP but not LTD of synaptic plasticity at PD 30 even if at the older age, when LTD was generally induced in the same-aged control rats. The loss of LTD in the mature DL striatum of adult animals has been reported to be accompanied by the motor abnormality [44]. This observation, together with the findings presented here, suggests that this abnormal synaptic plasticity in the DL striatum might be the important mechanism underlying movement disorders caused by in utero exposure to EtOH.

Among mechanisms responsible for the abnormal corticostriatal synaptic plasticity in PD 30 EtOH-exposed offspring, one might be that EtOH modulates glutamatergic neurotransmission either directly or via changing other neurotransmitter systems. Calabresi et al (1997) found that tetanic stimulation of corticostriatal afferent fibers produced NMDAr-dependent LTP in slices from D2R-null mice [30]. Based on this finding, D2R is considered to play a key role in controlling the direction of long-term changes in synaptic efficacy in striatum. However, a different viewpoint is raised here i.e. the appearance of LTP instead of LTD in EtOH offspring is caused through EtOH-induced up-regulation of D1R and down-regulation of D2R leading to the imbalance between the function of D1R and D2R. This conclusion is suggested by the following main findings. First, D2R antagonist L-sulpiride blocked LTD induction, but not facilitated LTP expression in control offspring. Second, D1R antagonist SCH23390 did not recover LTD though it blocked LTD induction in EtOH-exposed offspring. Third, SCH23390 could recover the basal synaptic transmission via eliminating the increase of presynaptic glutamate release induced by prenatal EtOH exposure. Forth, D2R agonist quiniride reversed NMDAr-dependent LTP into mGluR-dependent LTD via adjusting the balance between D1R and D2R in EtOH-exposed offspring. A large body of evidence has established that striatal LTD induction requires the activation of both D1R and D2R [16], [17], [45], whereas LTP expression is only related to D1R activation [26], [27]. And this was also proved by two main results acquired from the present study. One is that striatal LTD in control slices was blocked by inhibiting D1R or D2R. The other is HFS induced LTP in EtOH slices could be abolished by D1R antagonist but not D2R antagonist. It has been established striatal LTD shares several characteristics with other forms of synaptic plasticity in the brain [46]–[48]. The induction of striatal LTD is considered to be presynaptic through decreasing glutamate release, as shown by a decrease in the frequency but not the amplitude of spontaneouss EPSCs [49], [50] with an increase in PPF [50]. There is general agreement that D2R plays a negative role in regulating presynaptic glutamate release [39], [51]. The latest studies indicate that D2R activation evokes retrograde signal pathway via endocannabinoids leading to the reduction of glutamate release [18], [30], [49], [52]–[54]. The activation of cAMP-PKA signal pathway leading to the increase of presynaptic glutamate release is one of the important mechanisms involved in D1R-dependent LTP [26]. Our findings indicate that the imbalance between the function of D1R and D2R induced by up-regulation of D1R and down-regulation of D2R might be responsible for appearance of LTP instead of LTD in EtOH-exposed offspring. Earlier studies using other methods have proved that the exposure to high-dose EtOH during prenatal and postnatal development has long-lasting effects on central dopaminergic systems linked with behavioral rewarding effects. For example, microarray analysis has demonstrated that developmental EtOH exposure causes up-regulation of D1R in the rat or mouse striatum [5], [55]. Receptor binding study in rats treated prenatally with EtOH shows a significant reduction in functional D2R within the mature striatum [31], [56]. Of course, further study should be necessary preformed to determine the expression and function of D1R and D2R during the developmental striatum to support our conclusion. In addition, the cholinergic system is another important factor involved in stratial synaptic transmission and plasticity [53], [57]. Some reports have pointed out that a dysfunction of the hippocampal cholinergic system is one of important outputs for prenatal exposure to high-dose EtOH [58], [59]. Therefore, the impairment of cholinergic system induced by prenatal EtOH exposure might also mediate the abnormal synaptic plasticity of striatum. Although one of the conclusions in the present that prenatal EtOH exposure has a long-lasting effect on dopamine receptors is strongly supported by a large of increased literatures. To date, it is very difficult to explain the fact why the exposure to EtOH results in opposite changes of D1R and D2R function: an up-regulation of D1R function and a down-regulation of D2R function.

## Conclusions

The DL striatum controls motor activity by processing the flow of information arising from the cerebral cortex and projecting, *via* direct and indirect pathways, to the output nuclei of the basal ganglia [60]. Intrastriatal dopaminergic system takes part in this process by affecting the excitatory synaptic transmission and plasticity. Our results indicate a possible pathophysiologic mechanism underlying hyperlocomotion induced by prenatal EtOH exposure that imbalance in the function of D1R and D2R within the DL striatum impairs the development and maturation of corticostriatal synaptic plasticity.
